# Assessing care providers’ perceptions and beliefs about physical activity in infants and toddlers: baseline findings from the Baby NAP SACC study

**DOI:** 10.1186/s12889-015-1477-z

**Published:** 2015-02-07

**Authors:** Kathryn R Hesketh, Esther MF van Sluijs, Rachel E Blaine, Elsie M Taveras, Matthew W Gillman, Sara E Benjamin Neelon

**Affiliations:** Centre for Diet and Activity Research (CEDAR) and MRC Epidemiology Unit, University of Cambridge, Cambridge, UK; UCL Institute of Child Health, 30 Guilford Street, London, WC1N1EH UK; Department of Nutrition, Harvard School of Public Health, Boston, MA USA; Division of General Pediatrics, Pediatric Population Health Management, Mass General Hospital for Children, Boston, MA USA; Obesity Prevention Program, Department of Population Medicine, Harvard Medical School and Harvard Pilgrim Health Care Institute, Boston, MA USA; Department of Community and Family Medicine, Duke University Medical Center and Duke Global Health Institute, Durham, NC USA

**Keywords:** Infants, Toddlers, Physical activity, Child care, Baby NAPSACC

## Abstract

**Background:**

As children now spend increasing amounts of time in out-of-home care, care providers play an important role in promoting positive health behaviors. Little is currently known about providers’ perceptions and beliefs about physical activity, particularly for very young children. This study describes providers’ perceptions and beliefs about infants’ and toddlers’ physical activity, and assesses their knowledge of physical activity guidelines, to establish if and where providers may need support to promote physical activity in child care settings.

**Methods:**

We analyzed baseline data from a pilot randomized-controlled trial conducted in 32 child care centers in Massachusetts, USA. Providers completed physical activity-related questionnaires from which we compared twenty perception and belief questions for infant and toddler care providers.

**Results:**

203 care providers (96% female, mean ± SD age: 32.7 ± 11.2 years) from 29 centers completed questionnaires. A large proportion of providers (n = 114 (61.9%)) believed that infants should be active for 45 minutes or less each day, and only 56 providers (29.7%) perceived toddlers to require more than 90 minutes of activity per day. 97% of providers perceived it was their job to ensure children engaged in a healthy amount of physical activity and most (94.1%) perceived physical activity to be important to own their health, despite 13.3% finding it hard to find the energy to be physically active.

**Conclusions:**

This study is the first to assess the physical activity perceptions and attitudes of providers caring for infants and toddlers. Though all providers believed toddlers should engage in more physical activity than infants, most providers believed that young children require only a short amount of physical activity each day, below recommended guidelines. How provider perceptions influence children’s physical activity behavior requires investigation.

## Background

The early years represent a period of rapid growth and development [1] and are important for the initiation and development of positive health behaviors. Relatively little is known about how physical activity influences very young children’s health, yet emerging evidence suggests that even in the youngest age groups, physical activity is beneficial to children’s health and development [2].

Despite these potential benefits, physical activity guidelines for children under the age of 6 years have until recently been lacking [3]. In 2009, the National Association for Sport and Physical Education (NASPE) revised their recommendations [4,5] to provide physical activity guidelines for infants, toddlers, and preschool-aged children. These stated that infants should interact with caregivers in daily physical activities dedicated to exploring movement and the environment, be in environments to encourage and stimulate movement and active play for short periods of time several times a day, and have opportunities for structured and unstructured physical activity [4]. They also recommended toddlers engage in at least 30 minutes of structured and at least 60 minutes (but up to several hours) of unstructured physical activity per day [4].

Subsequent physical activity guidelines for children under 6 years have been released in Australia [6], the UK [7] and Canada [8]. In the US, the Institute of Medicine (IOM) has now specified four recommendations to increase physical activity and decrease sedentary time in infants, toddlers and pre-schoolers [1], with a particular focus on the child care environment. These guidelines encourage sufficient opportunities for children to be active throughout the day, an appropriate environment for physical activity, free from obstruction, and training for care providers providing advice to parents about increasing physical activity and decreasing sedentary time [1]. Additionally, the IOM recommended 15 minutes of moderate-to-vigorous physical activity for every hour in care [1].

Such recommendations are increasingly important, as many young children now spend the majority of their waking hours in out-of-home care [9]. In 2010, 35.3% of infants and 37.5% of toddlers with working mothers in the US were cared for by someone other than a relative [10], with the majority of these children being cared for in child care centers. These centers therefore serve as an important outlet for children’s physical activity [11], yet children appear to engage in little moderate to vigorous physical activity (MVPA) whilst in child care [12]. In general, young children appear to be sedentary for large proportions of time [13-15], and spend increasingly large amounts of time in screen-based activities [16], from a very young age [17]. Encouraging higher physical activity levels in the youngest age groups in these settings may therefore be beneficial for children’s development [1], and long-term health [2].

To date, the majority of research in the child care environment has focused on children older than 2 years, with little data available for infants and toddlers. Although average physical activity levels vary significantly between centers in preschool-aged children [18,19], they are likely to be more active in centers where greater opportunities to be active, both indoors and outdoors, are provided [20,21]. In one study, 2 and 3-year-olds with more opportunities to be active engaged in higher levels of physical activity [22]. Given the crucial role centers play in meeting children’s physical activity needs, they are a plausible and potentially successful intervention setting [23-25]. Interventions in older preschool children developed to increase physical activity by providing additional time [26], equipment [27] and provider training [28-30] have showed positive results, as has one providing training for care providers of toddlers and infants [31].

Despite promising intervention efforts, few have sought to harness the large influence that care providers have on children’s activity levels. As care providers may both encourage and discourage physical activity in preschool-aged children, consciously or otherwise [25], their perceptions, beliefs and own physical activity behaviors may be an important influence [32]. However, little is known about what factors might influence activity behavior in very young children, despite the developmental importance of physical activity in infants and toddlers [6-8] and the increasing amount of time they spend in child care [9]. Given the role of care providers in promoting physical activity in young children, and that strategies to promote physical activity are likely to differ depending on children’s age, this study assessed physical activity perceptions and beliefs of infant and toddler child care providers.

## Methods

### Participants and study design

We used baseline data collected in spring 2009 from a pilot randomized controlled trial intervention called the Baby Nutrition and Physical Activity Self-Assessment for Child Care (Baby NAP SACC). Full details of the study are presented elsewhere [33]. Briefly, the intervention aimed to help prevent obesity in children under the age of 2 years by improving policies and care provider practices related to nutrition and physical activity in child care centers. The 6-month-long intervention took place in a sample of 32 licensed centers (16 intervention, 16 control) serving racially and ethnically diverse children under the age of 2 years in the greater Boston, Massachusetts (MA) area [33]. Informed written consent was obtained from each center director prior to study onset, and the study was approved by the Human Subjects Committee of Harvard Pilgrim Health Care.

### Assessing provider physical activity perceptions and beliefs

All staff members, including directors and teachers (“providers”) involved in the care of infants and toddlers at the 32 study centers were asked to complete up to 3 self-administered written questionnaires.

All providers were first asked to complete a brief measure assessing their physical activity perceptions. Prior to study initiation, no instruments were available to assess provider perceptions of young children’s physical activity. These measures were therefore developed for the Baby NAP SACC study, tested with a small sample of providers, (n=2) to ensure face validity, and revised based on provider feedback.

We used providers’ responses to 12 statements (e.g. “Part of my job is making sure children get a healthy amount of physical activity”, see Table 1 for all 12 statements) to assess their perceptions about their role in supporting physical activity in young children (9 questions), and their own physical activity (3 questions) [34]. Statements relating to providers’ own physical activity were adapted from a questionnaire designed to assess physical activity, and barriers and facilitators to physical activity in adults [34]. Providers rated their agreement with each statement on a scale of 1 to 5 (1 = Strongly Disagree; 2 = Disagree 3 = Neutral, 4 = Agree, 5 = Strongly Agree). We then dichotomized these responses to determine whether providers agreed (strongly agree/agree) or did not (had no view/strongly/disagreed) with each statement.

**Table 1:** **Infant and toddler providers’ perceptions and beliefs about physical activity (derived from Baby NAPSACC questionnaires** ^**1,2**^ **)**

	**% providers strongly/agreeing with statement**
**Questions assessing providers’ perceptions about their role in supporting physical activity** ^**1**^	
Part of my job is making sure children get a healthy amount of physical activity	97.0
Teachers are very influential in helping children get a healthy amount of physical activity	94.6
I can get most children to be active during outdoor play and physical activities	93.1
I feel prepared to encourage children to be physically active	92.1
An important aspect of my work is providing children with opportunities for physical activity	91.0
When I make an effort to encourage children to be active, they get a lot of physical activity	90.1
Teachers are well suited to promote physical activity in young children	87.6
Teachers have limited responsibility to promote physical activity in young children	22.8
Encouraging adequate physical activity in children is not the responsibility of the teacher	7.9
**Questions assessing providers’ perceptions about their own physical activity levels** ^**1**^	
Physical activity is important to my own health	94.1
I find it hard to work up the energy for my own physical activity	13.3
Physical activity is low on the list of things I want to do	7.4
**Questions assessing providers’ beliefs about physical activity in infants and toddlers** ^**2**^	
It is important for < I/T > to learn new physical skills	91.3
It is important for < I/T > to be physically active from an early age	88.2
Being physically active as an < I/T > can help prevent too much weight gain	72.1
A physically active < I/T > tends to make the house messy	25.3
A physically active < I/T > is likely to hurt him/herself easily	20.5
<I/T > should learn to be still in public places at an early age	19.3
I can attend to my other responsibilities better if I do not have to worry about < I/T > moving around	13.8
It is difficult to manage a physically active < I/T>	11.2
A physically active < I/T > is likely to get into trouble	10.6

^1^Provider perceptions about their role in supporting physical activity in young children (9 questions), and their own physical activity (3 questions) [34] (n = 203); ^2^Providers’ beliefs about physical activity in infants and toddlers [35] (n = 161); I: Infant; T: Toddler.

We used 2 questions, again developed for the Baby NAP SACC study, to assess provider knowledge of the daily activity recommendations for infants and toddlers. Providers were asked “How many minutes of physical activity should infants 0-12 months of age get each day?” and “How many minutes of physical activity should toddlers 12-24 months of age get each day?” Providers chose one of 8 categories (0 minutes; 1-15; 16-30; 31-45; 46-60; 61-90; 91-120; 120-150 minutes) in response to each question.

Providers were also asked to complete a questionnaire related to their beliefs about physical activity for infants, toddler, or both, depending on their assigned classroom in the center. These questions were based on an instrument available and in use at the time of the Baby NAP SACC study [35] to assess parental beliefs about physical activity. We used the version of the instrument that included questions related to both infant and toddler physical activity and feeding behavior, although the final questionnaire focused solely on feeding behaviors [35]. As these questionnaires were originally designed to assess parental beliefs and behaviors, we adapted the wording for some questions to be used with care providers. For example, we changed “your child” to “the children in your care”, “infants” or “toddlers” where relevant. We used 8 statements (which were identical save for the word ‘infant’ or ‘toddler’) to assess provider beliefs about physical activity in young children (e.g. “Being physically active as an infant/toddler can help prevent too much weight gain”, see Table 1 for all 8 statements). Providers responded to each statement on a 5-point likert scale (1 = Disagree; 3 = Neutral, 5 = Agree), and we dichotomized their responses to determine whether providers agreed (4/5) or did not (1-3) with each statement. These measures had acceptable reliability (Cronbach's alpha of 0.69 for infant questionnaire and 0.77 for the toddler questionnaire).

All care providers reported their age, sex, role within the center, education level, and race and ethnicity. Care providers stated how long they had worked in child care in general. Center directors were also asked to provide basic center demographic information, including the number of years the center had been in operation; the number of children enrolled; the number of care providers in the center; participation in the Child and Adult Care Food Program (CACFP); and National Association for the Education of Young Children (NAEYC) accreditation.

### Analysis

Data were analyzed using STATA/IC 12. Basic descriptive statistics were calculated for providers participating in the study and for the centers from which they were recruited. Missing data accounted for fewer than 3% of observations, with only provider age (13%) and time spent at current center (10%) missing in ≥10% of participants.

We calculated the percentage of providers agreeing with each of the 20 statements relating to their perceptions of supporting physical activity in young children, their own physical activity and their beliefs about physical activity in young children. We also generated descriptive data about provider perceptions of the recommended levels of physical activity for infants and toddlers. To explore differences in infant/ toddler provider beliefs about physical activity, we compared provider responses by type (infant/toddler) using χ^2^ tests. A significance level of α = 0.05 was set *a priori.*

## Results

Baseline data were available from 29 child care centers (Intervention: n = 14; Control n = 15), as 3 centers withdrew from the study prior to completing the measures. Of the 203 child care providers who participated, 191 were female (96%), with a mean age (SD) was 32.9 (11.1) years. Centers from which these participants were drawn had been in operation for a median (Interquartile range) of 13 (10-18.2) years, had 63 (35-111) children enrolled, with 13 (10-24) care providers per center. 21 centers (72.4%) participated in CACFP and 16 centers (55.2%) were accredited by the NAEYC.

The majority of providers perceived they had a role in supporting physical activity in young children (Table 1), although 22.8% of providers believed they had limited responsibility to promote physical activity in young children. Most providers (94.1%) perceived physical activity to be important to their health, despite 13.3% finding it hard to find the energy to be physically active. Just under three quarters of providers (72.1%) believed that physical activity could prevent too much weight gain in infants and toddlers; toddler providers were more likely to believe this than infant providers (80.2 vs. 56.4%, Chi^2^*p* < 0.001). Compared to infant providers, toddler providers were more likely to believe that toddlers should learn to be still in public places at an early age (24.5 vs. 9.1%, Chi^2^*p* = 0.01).

The amount of activity providers perceived infants and toddlers to require each day is shown in Table 2. A large proportion of providers (n = 114 (61.9%)) believed that infants should be active for 45 minutes or less each day, and only 56 providers (29.7%) perceived toddlers to require more than 90 minutes of activity per day. All care providers believed that toddlers should engage in more activity each day than infants.

**Table 2:** **Provider-perceived daily activity requirements for infants and toddlers**

**Perceived daily activity requirements**	**Number of providers (%)**	
	**For infants** ^**1**^	**For toddlers** ^**2**^
1-15 minutes	27 (14.7)	0 (0)
16-30 minutes	47 (25.5)	19 (10.1)
31-45 minutes	40 (21.7)	32 (16.9)
46-60 minutes	34 (18.5)	52 (27.5)
61-90 minutes	17 (9.2)	30 (15.9)
91-120 minutes	6 (3.3)	30 (15.9)
121-150 minutes	13 (7.1)	26 (13.8)

^1^184 infant care providers answered the question: “How many minutes of physical activity should infants 0-12 months of age get each day?”; ^2^189 toddler care providers answered the question: “How many minutes of physical activity should toddlers 12-24 months of age get each day?”.

## Discussion

This study is the first to assess the physical activity perceptions and attitudes of providers caring for infants and toddlers. Providers, regardless of whether they cared for infants or toddlers, were positive about their role in supporting physical activity. Just under a quarter did however believe they had limited responsibility to promote physical activity in young children, and over 10% of providers found it hard to work up the energy for their own physical activity. A majority of providers believed that infants required less than one hour of physical activity each day, and less than 45% of providers correctly stated that infants should be active for at least 90 minutes per day (the physical activity guidelines in use at the time of data collection [4]). All providers believed toddlers required more physical activity than infants. Ensuring providers are aware of and encourage children to achieve the recommended daily amounts of physical activity [1,36] may be important to support and promote health and development of very young children [2].

Providers were generally positive about their role in supporting physical activity, with many agreeing with statements relating to their influence on children’s activity (e.g. when I make an effort to encourage children to be active, they get a lot of physical activity; I can get most children to be active during outdoor play and physical activities). These results fit with previous qualitative and quantitative literature conducted in preschool-aged children, which suggests that providers act as ‘gate-keepers’ [37], both facilitating [37-39] and impeding children’s physical activity when in care [37,40]. Moreover, providers’ own behaviors have been shown to influence physical activity levels in preschoolers previously [25]. Given that a number of providers reported finding it hard to find the energy for their own physical activity here [25,37], more work is required to establish how providers’ own activity behaviors are associated with those of the young children in their care.

Almost a quarter of providers (22.8%) perceived they had limited responsibility to promote physical activity in young children. This may be due to providers’ perceptions that young children naturally engage in high levels of activity [41,42] and do not therefore require encouragement. With many competing demands on providers’ time, they may also choose not prioritize physical activity, particularly when faced with the need to ensure children are meeting cognitive milestones [11]. The social expectation that toddlers should sit still was also apparent here. As seen in parents [43] and providers [42] caring for preschool-aged children previously, these findings highlight for the first time in younger children the tension between the expectation that children should be ‘well behaved’ and the need for sufficient physical activity.

In order to promote physical activity in young children, it is important that providers are aware of the amount of physical activity infants and toddlers require. Here, most providers believed less than one hour of physical activity per day was sufficient for infants, and fewer than half of providers correctly stated that toddlers should be active for at least 90 minutes on average per day (the NASPE physical activity recommendations in place at the time of data collection in 2009 [4]). More recent activity guidelines for children under 6 years, issued in Australia [6], the UK [7] and Canada [8], advocate 3 hours of activity during waking hours for children who are able to walk, with specification of age-appropriate guidelines such as encouraging tummy time for non-walkers [6-8]. The US Institute of Medicine (IOM) suggests young children engage in 15 minutes of activity in every waking hour in care [1], which also equates to approximately 2 hours of activity over an 8-hour day in care. Though evidence to support these guidelines, particularly with regards to intensity of activity [44], is currently sparse [6], the field is moving towards a consensus with regards activity requirements in young children. Simple interventions to boost providers’ knowledge, confidence and ability to provide sufficient active opportunities [33] whilst children are in care are therefore likely to be beneficial for infants and toddlers [2].

As children spend increasingly large periods of time in child care [9], they and their parents rely on providers to allow for sufficient opportunities to be active [11,41]. Most providers believed that it was important for children to be physically active from an early age, and for infants and toddlers to learn new physical skills. Work should now determine whether these perceptions influence physical activity levels and developmental outcomes of children in their care. Moreover, establishing how providers’ own behaviors are associated with children’s in-care physical activity would be beneficial, to determine if using providers as positive role models may enhance interventions to increase physical activity in child care settings.

### Strengths and limitations

This study was conducted in a racially and ethnically diverse sample, using existing questionnaires to assess care providers perceptions and beliefs. Despite the majority of providers being white females, there was a range of educational attainment across providers, which is likely to reflect the profession as a whole. Providers had varied experience both within their current center and in child care more generally. Although it would have been interesting to assess clustering of provider responses within centers, this was not possible due to the small numbers of providers in some centers (Range: 1-20).

It is possible that asking providers about their perceptions and beliefs about physical activity may have introduced a social desirability bias, particularly in light of the wider aims of Baby NAP SACC to improve the nutrition and physical activity environments of child care centers. Although perceptions and beliefs were assessed at baseline before the intervention had taken place, the majority of providers tended to agree with more socially desirable responses, and disagree with less socially desirable ones. The questions relating to children’s physical activity requirements are however less likely to be influenced by social desirability, as there was not an obvious “correct” answer. Therefore, responses may be seen as more of a true representation of provider knowledge. This paper provides a baseline indication of infant and toddler providers’ views about physical activity. Unfortunately, objective measurement of physical activity was not undertaken in this study, as it would have been preferable to assess providers’ own physical activity behaviors and children’s physical activity levels within centers to determine how these relate to provider perceptions and beliefs. In addition, as participants were drawn from a small region within one state, further work is needed to assess how perceptions vary both within and across states, and between countries.

## Conclusions

Despite care providers’ perceptions that they play a role in supporting young children’s physical activity, many believe young children require less activity than is currently recommended. As increasing numbers of children spend their waking hours in child care, providers may be important in influencing children’s activity behavior. Future work should determine what influence providers’ beliefs and perceptions, and their own physical activity, have on young children’s physical activity behavior. This is necessary to determine whether harnessing the large potential influence of child care providers may enhance interventions to promote physical activity in very young children.

## Abbreviations

Baby NAP SACC: Baby Nutrition and Physical Activity Self-assessment for Child Care
US: United States
NASPE: National Association for Sport and Physical Education
UK: United Kingdom
IOM: Institute of Medicine
